# Complete Genome Sequence of Lactobacillus plantarum Strain LQ80, Selected for Preparation of Fermented Liquid Feed for Pigs

**DOI:** 10.1128/genomeA.00530-18

**Published:** 2018-06-21

**Authors:** Naoko Moriya, Kazuma Nakano, Akino Shiroma, Misuzu Shinzato, Noriko Ashimine, Maiko Minami, Hinako Tamotsu, Makiko Shimoji, Tetsuhiro Nakanishi, Shun Ohki, Kuniko Teruya, Kazuhito Satou, Takashi Hirano, Tatsuro Hagi, Miho Kobayashi, Masaru Nomura, Hiromi Kimoto-Nira, Kiyoshi Tajima, Yimin Cai, Chise Suzuki

**Affiliations:** a Institute of Livestock and Grassland Science, NARO, Tsukuba, Ibaraki, Japan; b Okinawa Institute of Advanced Sciences, Uruma, Okinawa, Japan; c Japan International Research Center for Agricultural Sciences, Tsukuba, Ibaraki, Japan

## Abstract

Lactobacillus plantarum LQ80 is a strain isolated from liquid feed for pigs. We determined the complete genome sequence of this strain using the PacBio RS II platform. LQ80 contained a single circular chromosome of 3,230,192 bp, with 44.66% G+C content and seven plasmids.

## GENOME ANNOUNCEMENT

Lactobacillus plantarum is found in various environmental niches, e.g., vegetable pickles (1–3), fermented milk products (4), and human gastrointestinal tracts (5). Strains of L. plantarum are considered to be useful starters not only in the food industry, but also for the preparation of silage (6) and fermented liquid feed (7–9) because of their great acidification ability via the production of lactic acid. Furthermore, some strains of L. plantarum are tolerant to gastric juice and bile acid (10) and are generally considered probiotics (11, 12).

L. plantarum LQ80, isolated from liquid feed for pigs, enhances the immune response (13) and the growth of intestinal villi (14) in weaning piglets. Fermented liquid feed prepared with LQ80 enhances bacterial diversity in the digestive tract of piglets (15). Genomic information for this strain would help clarify these potential functions as probiotic lactic acid bacteria. Therefore, we report the genome sequence of strain LQ80.

Strain LQ80 was cultured with de Man-Rogosa-Sharpe medium at 37°C. For the genome sequencing of LQ80, DNA was isolated at the early log phase. DNA was purified using a PowerClean DNA cleanup kit (Mo Bio Laboratories, Carlsbad, CA), followed by 20-kb library construction for P6-C4 chemistry with shearing. Five single-molecule real-time (SMRT) cells were used for sequencing on the PacBio RS II platform (Pacific Biosciences, Menlo Park, CA) with a 180-min movie time. We obtained 320,152 reads with a mean length of 5,268 bp. *De novo* assembly was performed using the Hierarchical Genome Assembly Process version 3 (HGAP3) (16). Four circular contigs were obtained, representing one chromosome (3.2 Mb) and three plasmids (92, 58, and 30 kb). For sequencing plasmids smaller than 30 kb, plasmid DNA was extracted at the stationary phase per the Qiagen user-developed protocol (see https://www.qiagen.com/us/resources/resourcedetail?id=8b1b2b4f-3dca-4447-8d21-f6fd64c3a729), and a 5-kb library was constructed with shearing. One SMRT cell was used for sequencing on the PacBio RS II platform with a 360-min movie time. The result of 362,945 reads with a mean length of 1,519 bp was assembled with HGAP3, and five circular contigs were obtained, representing five plasmids (30, 16, 10, 6, and 3.5 kb). From this analysis, another four smaller plasmids were constructed.

The LQ80 chromosome was 3,230,192 bp in length, with a G+C content of 44.66%. In addition, LQ80 had seven plasmids: pLQ801 (92,191 bp), pLQ802 (58,027 bp), pLQ803 (30,644 bp), pLQ804 (16,405 bp), pLQ805 (10,218 bp), pLQ806 (6,355 bp), and pLQ807 (3,592 bp). The complete genome was annotated using NCBI Prokaryotic Genome Annotation Pipeline (PGAP) (17) and Microbial Genome Annotation Pipeline (MiGAP) (18). PGAP identified 3,371 genes, including 3,186 coding sequences, 88 RNAs, and 97 pseudogenes. MiGAP identified 3,260 coding sequences and 88 RNAs. Plasmids pLQ801, pLQ802, pLQ803, pLQ804, pLQ805, and pLQ807 contained candidate genes for the plasmid replication initiation proteins.

### Accession number(s).

The complete genome sequences of the chromosome and seven plasmids of L. plantarum LQ80 have been deposited in GenBank under accession numbers CP028977 (chromosome), CP028978 (pLQ801), CP028979 (pLQ802), CP028980 (pLQ803), CP028981 (pLQ804), CP028982 (pLQ805), CP028983 (pLQ806), and CP028984 (pLQ807).
